# Lead contamination of public drinking water and academic achievements among children in Massachusetts: a panel study

**DOI:** 10.1186/s12889-021-12474-1

**Published:** 2022-01-15

**Authors:** Wenxin Lu, Ronnie Levin, Joel Schwartz

**Affiliations:** Department of Environmental Health, Harvard T.H. Chan School of Public Health, Boston, USA

**Keywords:** Environmental epidemiology, Lead, Water, Children, Education, Cognitive development, Academic performance

## Abstract

**Background:**

Public drinking water can be an important source exposure to lead, which can affect children’s cognitive development and academic performance. Few studies have looked at the impact of lead exposures from community water supplies or their impact on school achievements. We examined the association between annual community water lead levels (WLLs) and children’s academic performances at the school district level.

**Methods:**

We matched the 90th percentile WLLs with the grade 3–8 standardized test scores from the Stanford Education Data Archive on Geographic School Districts by geographic location and year. We used multivariate linear regression and adjusted for urbanicity, race, socioeconomic characteristics, school district, grade, and year. We also explored potential effect measure modifications and lag effects.

**Results:**

After adjusting for potential confounders, a 5 μg/L increase in 90th percentile WLLs in a GSD was associated with a 0.00684 [0.00021, 0.01348] standard deviation decrease in the average math test score in the same year. No association was found for English Language Arts.

**Conclusions:**

We found an association between the annual fluctuation of WLLs and math test scores in Massachusetts school districts, after adjusting for confounding by urbanicity, race, socioeconomic factors, school district, grade, and year. The implications of a detectable effect of WLLs on academic performance even at the modest levels evident in MA are significant and timely. Persistent efforts should be made to further reduce lead in drinking water.

**Supplementary Information:**

The online version contains supplementary material available at 10.1186/s12889-021-12474-1.

## Background

Lead is a prevalent environmental contaminant and a known neurotoxic agent. Lead crosses the blood brain barrier [1], interferes with the calcium-regulated release of neurotransmitters [2] and induces programmed cell death of the nervous system [3]. Developing brains of children are particularly vulnerable to the neurotoxic effects of lead. Exposure to even low levels of lead shows evidence of long-term damage to children’s cognitive function and IQ [4–10], and children’s academic performance [11–18], with implications for future academic and career achievements [19, 20].

Despite widespread reported compliance with EPA’s 1991 Lead and Copper Rule (LCR), as administered by state drinking water agencies, drinking water remains an important potential lead exposure source [21–23]. Lead rarely occurs in natural water sources, it contaminates drinking water via the corrosion of lead pipes, solder, faucets, cisterns, and other plumbing components containing lead. Exposure to lead from drinking water has been associated with variabilities in children’s blood lead levels (BLLs) [24–27]. Interventions, such as lead pipe replacement, can significantly reduce WLLs [28], and consequently, BLLs [29]. EPA estimated that drinking water generally constitutes more than 20% of average daily lead exposure, 40 to 60% for infants who consume mostly infant formula (dry powder or liquid concentrate) mixed with tap water, and up to 80% of children’s daily exposure in some realistic circumstances even in public water supplies (PWSs) that are not exceeding EPA’s LCR [30]. Variability, especially in water sample collection methods, complicates comparing studies on the relationship between BLLs and WLLs [22]. Quantifying the contribution of WLLs to BLLs in children is further complicated because of the difficulty of collecting reproducible water lead samples; relatively small fluctuations in factors such as temperature, pH, alkalinity, and dissolved solids affect the solubility of lead [31, 32].

Exposure to other sources of lead, such as the legacy lead deposition in soils, have been found to affect the cognitive abilities of young children [33]. Yet few studies have attempted to directly quantify the health impact of WLLs. We investigated whether water system wide WLLs are reflected in the academic performances of children on a population level, without information on individual blood lead concentrations. We found only one study investigating a short-term water-lead exposure with school performance [34], but that was unpublished.

Most previous studies relating lead exposure with child academic performances focused on long-term cumulative effects. For instance, Miranda et al. found that BLLs in early childhood (0–5 years old) were associated with lower 4th grade math and reading test scores [35]. Reyes showed that children in Massachusetts with higher BLLs in early childhood (before age 6) perform worse in standardized tests in grades 3 and 4 [36]. Aizer et al. showed that reducing BLLs before the age of 6 reduces the probability of low reading and math proficiency at 3rd grade [37]. A New York study found county-level incidence of higher BLLs is associated with lower test scores 3–7 years later in grades 3–8 [13]. To the best of our knowledge, no one has investigated the potential impact of recent water lead exposures on cognitive function and academic performance.

To examine whether recent WLLs are associated with children’s test scores on the population level, we matched the community WLLs to the test scores of students from grade 3 to grade 8 in Massachusetts by school district. We constructed a panel dataset using repeated measurements from 2010 to 2016, and tested math and English Language Arts (ELA) separately. We adjusted for urbanicity, race, SES and other potential confounders.

## Methods

### Study Population

The study region is the Commonwealth of Massachusetts, USA, which is partitioned into 294 Geographic School Districts (GSD). GSDs with incomplete information on geographical coverage were removed. We restricted to GSDs that are served by at least one Community Water System (CWS), i.e., a public water system that supplies water year-round to at least 25 people at their primary residence or at least 15 primary residences [38]. Standardized test score data and lead concentration data were available for the remaining 229 GSDs from 2009 to 2016. Because GSD-level covariates data are generally missing for year 2009, we restricted to the 7-year period from 2010 to 2016, constituting 2,164,208 student-years during the period. To test whether restricting to the 229 GSDs resulted in any selection bias, we compared the average test score among the 229 included GSDs and the 65 excluded GSDs. The average standardized test scores pooled across subjects are 0.482 and 0.479 for the included and excluded GSDs, respectively. A two-sample t-test showed no significant difference between the two groups [*p* = 0.95]. Since the outcome is uncorrelated with whether the GSD was included in the analysis, the possibility of selection bias is minimal.

### Lead concentration data

The 90th percentile lead concentration data in each CWS was obtained from the MA Department of Environmental Protection (DEP), which is the primacy agency for the state of MA under the Safe Drinking Water Act. The federal LCR requires, among other conditions, that WLLs in a CWS not exceed the Action Level (AL) of 15 μg/L at the 90th percentile across all samples in a monitoring period [30].

Under the LCR, each CWS must submit two documents for DEP approval before sampling begins: A Materials Evaluation that surveys the material and structure characteristics of a pool of target sampling sites; and a Sampling Plan that identifies the sampling sites at the highest risks of elevated lead concentrations based on their structure characteristics, including the likelihood of lead service lines, solder, and goosenecks. The majority of sampling sites for CWSs are households, including single-family and multi-family residences. Under the federal LCR, the water sample from each residence in the sample pool is a one liter first-flush sample taken after a minimum 6-h stagnation interval to approximate the routine high exposures that can occur in each home every morning and/or at the end of the day when residents return home from work or school. Lead analysis is conducted at certified laboratories. The sample lead concentrations are reported to the DEP and the 90th percentile lead concentrations for each CWS are recorded. During each monitoring period, the CWS must collect a prescribed number of 1-l first-draw water samples from their available sampling sites. The number of sampling sites required depends on the size of the population served (Table 1) [39].

**Table 1:** Number of required sampling sites required for community water system sizes, for standard monitoring (semi-annual) and reduced monitoring (annual and triennial)

Size of CWS (People served)	Number of sampling sites (standard monitoring)	Number of sampling sites (reduced monitoring)
> 100,000	100	50
10,001 – 100,000	60	30
3301 – 10,000	40	20
501–3300	20	10
101–500	10	5
≤ 100	5	5

Also, under the LCR, there are 3 monitoring periods annually: Q2 (Jan 1st to Jun 30th), Q3 (Jun 1st to Sep 30th) and Q4 (Jul 1st to Dec 31st). The standard testing schedule for CWSs is semiannual testing, which happens in Q2 and Q4 each year. If a CWS has met both the lead and copper requirements for two consecutive semi-annual monitoring periods, monitoring frequency is reduced from semi-annual to annual. If a CWS has met both the lead and copper requirements for three consecutive annual monitoring periods, its monitoring frequency can be further reduced from annual to triennial. If the lead or copper requirement is violated during a reduced-frequency monitoring period, the monitoring frequency is reversed to semiannual. An increase in monitoring frequencies can also result from changes in source water, treatment methods and overall operations of the water systems, or failing to operate within their water quality parameters for more than nine days in any six-month period. When the LCR is violated, the CWSs are required to undergo interventions such as corrosion control treatment, lead service line replacement and public education. Systems under annual and triennial testing sample during the monitoring period Q3, and the number of sampling sites required in these systems are also reduced, as described in Table 1. The requirement that reduced sampling should be implemented during summer months was proposed in the Preamble to the 1991 LCR, because plumbosolvency is increased by temperature, resulting in higher WLLs in the summer [32, 40].

The MA Water Resources Authority (MWRA) is the largest water supplier in MA, serving approximately 2,222,151 people in 2010 [41]. For CWSs served by the MWRA, the Q3 monitoring period is from July 1st to October 31st, which is slightly different from the Q3 monitoring period of other CWSs independent of MWRA. Of the 351 cities and towns in Massachusetts, 32 cities and towns, including the most populous city Boston, are fully supplied by MWRA; another 15 cities and towns are partially supplied by MWRA [42].

The DEP data cover 438 active CWSs with 1847 drinking water sources, serving a total of approximately 4,819,215 people. As a result of the different sampling frequencies, the monitoring periods in the data are of varying lengths. Among all MA CWSs from 2010 to 2016, there were 408 semi-annual monitoring periods, 446 annual monitoring periods, 1180 triennial monitoring periods and 10 monitoring periods of other lengths. About 95.1% of the data were collected in the Q3 (summer) monitoring period. To address monitoring periods of varying lengths, we calculated the 90th percentile WLL for each CWS-year as a weighted average of the 90th percentile lead concentrations of all monitoring periods in that year, weighted on the number of days in the monitoring periods covered in that year. For this study, we assumed the 90th percentile WLLs are indicative of the relative ranking of community exposure. Similar WLL data and assumptions have been used in other published studies [43, 44]. For simplicity, we will refer to the 90th percentile WLL as the WLL in this analysis. The WLL for each GSD-year was calculated as the average of the WLLs of the active CWSs located within the GSD, weighted on the population served by each CWS site. The geographic location of the CWS sites was retrieved from the MA Bureau of Geographic Information (MASSGIS) [45]. Data on population served by each CWS was retrieved from the Environmental Working Group’s Tap Water Database [46].

### Test score and covariates

The GSD level standardized test score data was retrieved from the Stanford Education Data Archive (SEDA) version 3.0 [47]. Individual-level data were not available for the protection of the privacy of student educational records, required by The Family Educational Rights and Privacy Act (FERPA) (20 U.S.C. § 1232 g; 34 CFR Part 99) [48]. We chose data aggregated on the GSD-level because it was the highest resolution with annual data available. The data we obtained contains GSD-level academic achievement information measured as standardized test scores in Mathematics and ELA, which in MA is the Massachusetts Comprehensive Assessment System (MCAS). The scale of the student test scores can be interpreted as the number of standard deviations (SD) above the average student performance, compared to a reference cohort (students in the 4th grade in 2009). For instance, a GSD-grade with a 0.5 average standardized test score indicates that the students of that grade in that GSD performed on average 0.5 SD higher than the average test score of the reference cohort in the same grade. The standardized test score for all GSDs in the US follows an approximate normal distribution. In this study, we included only GSDs in MA. The median MA standardized test score is approximately 0.49 for math and 0.47 for ELA, indicating that MA students perform above the national average by grade. Data are available for each grade-year-subject from grade 3 to grade 8 and from 2008 to 09 to 2015–16 school years. After restricting to the 229 GSDs with complete geographic information and served by at least one CWS, the remaining dataset covers 2,164,028 enrolled student-years and 5,677,721 tests. For each enrolled student-year, two tests are administered (math test and ELA test), thus the number of tests is approximately twice the number of student-years. The actual ratio of tests versus student-years is slightly higher than 2, due to practical arrangements such as retests.

Covariates data with the SEDA datasets are from the Common Core of Data of the National Center for Education Statistics and the American Community Survey. The covariates included in the models in this study include four categories: (1) urbanicity: urban, sub-urban, town and rural; (2) GSD SES characteristics: log of median income, bachelor’s degree rate, poverty rate, Supplemental Nutrition Assistance Program (SNAP) recipient rate, single-mother household rate and unemployment rate; (3) student racial composition: proportion of native Americans, Asians, Hispanics, Blacks and Whites; and (4) student SES characteristics: proportion of English Language Learners (ELL), proportion of reduced-price lunch eligible students and proportion of economically disadvantaged students.

### Statistical analysis

We used a multivariate linear regression with fixed effects for time and cohort, which is a variant of the causal Difference-in-Difference (DID) analysis [49–51]. In this study, a cohort is defined as all students enrolled in a specific grade in a specific GSD, and time is school year. This model controls for unmeasured confounders across GSDs, grades within GSDs, and time periods by adding dummy variables for each cohort and year [52]. Fixed or slowly varying covariates within GSD are also removed by the cohort dummy variables. The exposure of interest is WLL for each cohort within each year; the outcome of interest is cohort-year standardized test scores. Thus, after controlling for cohort fixed effects and year fixed effects, we tested whether deviations in WLL from the cohort-year average are associated with deviations in standardized test scores from the cohort-year average.

The base model takes the following form:

$${\mathrm{S}}_{\mathrm{itg}}={\beta}_1{WLL}_{it}+{\beta}_2{Z}_{it g}+{\alpha}_{ig}+{\gamma}_t+{\epsilon}_{it g}$$

where i indexes GSD, t indexes school year and g indexes grade. The dependent variable S_itg_ is the standardized test score for grade g students in GSD i in year t. The independent variable of interest WLLi_t_ is the average WLL in GSD i in year t. Z_itg_ are cohort-level covariates associated with academic performances for grade g students in GSD i in year t. *α*_*ig*_ are cohort fixed effects for GSD i and grade g, and *γ*_*t*_ are year fixed effects for year t. Because Z_itg_ are time varying, adjusting for these covariates controls for differences across GSDs due to differential changes of these covariates over time. We used linear models in panel data in R version 4.0.5 (package “plm”).

This model is generalized from a traditional DID model by extending the scope to more than two time periods and investigating a continuous treatment variable. The continuous treatment is the change in WLL for a GSD over two consecutive years, and the outcome is the change in standardized test scores for a GSD over the same two years. Consider two cohorts A and B over two consecutive time periods t and t + 1. Assume the exposure level of cohort A does not change from t to t + 1, while the exposure level of cohort B increased by 1 unit from t to t + 1. In this scenario, cohort A can be considered a negative outcome control for cohort B: changes in the outcomes in cohort A cannot be due to variations in the exposure levels, and can only be explained by variations in the same set of covariates as cohort B. For both cohorts A and B, the outcome levels over the two time periods, the expected differences, and DID estimates are presented in Table 2. The linear DID estimate between the two cohorts over the two time periods is:

$${\beta}_1+{\beta}_2\left[\left({Z}_{B,t+1}-{Z}_{Bt}\right)-\left({Z}_{A.t+1}-{Z}_{At}\right)\right]$$

Under the assumption that the time varying covariates Z_itg_ capture all other factors affecting the trend of the outcome and that there are no interactions between cohort fixed effects and year fixed effects [53], the estimated coefficient β_1_ is an estimate of the causal treatment effect of a 1-unit increase of WLL over the time periods t to t + 1. This method was used in a recent paper investigating the association between ambient air pollution and children’s academic performances in the US [54].

**Table 2:** linear DID estimation process with two-way fixed effects models

	Cohort A (no change in WLL)	Cohort B (WLL increased by 1 unit)
t	*β*_1_*WLL*_*At*_ + *β*_2_*Z*_*At*_ + *α*_*A*_ + *γ*_*t*_ + *ϵ*_*At*_	*β*_1_*WLL*_*Bt*_ + *β*_2_*Z*_*Bt*_ + *α*_*B*_ + *γ*_*t*_ + *ϵ*_*Bt*_
t + 1	*β*_1_*WLL*_*At*_ + *β*_2_*Z*_*A*, *t* + 1_ + *α*_*A*_ + *γ*_*t* + 1_ + *ϵ*_*A*, *t* + 1_	*β*_1_(*WLL*_*Bt*_ + 1) + *β*_2_*Z*_*B*, *t* + 1_ + *α*_*B*_ + *γ*_*t* + 1_ + *ϵ*_*B*, *t* + 1_
E(Difference)	*β*_2_(*Z*_*A*, *t* + 1_ − *Z*_*At*_) + (*γ*_*t* + 1_ − *γ*_*t*_)	*β*_1_ + *β*_2_(*Z*_*B*, *t* + 1_ − *Z*_*Bt*_) + (*γ*_*t* + 1_ − *γ*_*t*_)
DID	*β*_1_ + *β*_2_[(*Z*_*B*, *t* + 1_ − *Z*_*Bt*_) − (*Z*_*A*. *t* + 1_ − *Z*_*At*_)]	

To test the potential lag effects of WLLs, we replaced the WLLs in the model with the WLL level from the previous year. The model takes the following form:

$${\mathrm{S}}_{\mathrm{itg}}={\beta}_1{WLL}_{i,t-1}+{\beta}_2{Z}_{itg}+{\alpha}_{ig}+{\gamma}_t+{\epsilon}_{itg}$$

Where WLL_i,t-1_ is the average WLL in GSD i in year t-1. The coefficient β_1_ and its statistical significance is compared with the respective coefficient from the results of the base model.

To test potential effect measure modifications by grade and racial composition, we modified the base model by adding an interaction term between the WLLs and the potential effect modifier. The model takes the following form:

$${\mathrm{S}}_{\mathrm{itg}}={\beta}_1{WLL}_{it}+{\beta}_2{WLL}_{it}{Y}_{it g}+{\beta}_3{Z}_{it g}+{\alpha}_{ig}+{\gamma}_t+{\epsilon}_{it g}$$

Where Y_itg_ is the level of the potential effect modifier of grade g students in GSD i in year t. The coefficient β_2_ is tested for statistical significance. A deviation of β_2_ from zero implies that the association between WLL and test scores is different among observations with different levels of the effect modifier Y_itg_.

Because the number of children in each grade in each school districts varies substantially, we used weighted least squares (WLS), where the weight is the number of students enrolled in each cohort within each year. We truncated the weights at the 5th and 95th percentiles to avoid influential observations and to stabilize the weighting. The weights for cohorts with number of enrolled students more than the 95th percentile of all cohorts were replaced by the 95th percentile, which is 596 students; the weights for cohorts with number of enrolled students less than the 5th percentile of all cohorts were replaced by the 5th percentile, which is 25 students.

## Results

### Summary Statistics

Table 3 presents the summary statistics of standardized test scores, number of student enrollments, average WLLs and socioeconomic characteristics of the 229 GSDs in Massachusetts, averaged across grades 3–8 and years 2010–2016. Urbanicity and GSD SES characteristics vary by district and year, while student racial composition and student SES characteristics vary by district, year and grade. The median standardized math and ELA test scores in Massachusetts are 0.4949 and 0.4672 SD above the national median level, respectively.

**Table 3:** GSD-level summary statistics, averaged across grades and years

	Min	25th percentile	Median	Mean	75th percentile	Max	SD	n
Standardized Math Test Score^a^	−0.417	0.314	0.495	0.495	0.696	1.266	0.299	228
Standardized ELA Test Score^a^	−0.555	0.284	0.467	0.467	0.654	1.170	0.293	228
Number of enrolled students	10.21	96.00	181.19	229.43	289.24	1911	224.45	229
WLLs (μg/L)	0.143	2.349	3.960	4.433	5.793	27.279	3.172	222
Percentage Native American (%)	0.000	0.047	0.136	0.256	0.265	5.568	0.513	229
Percentage Asian (%)	0.000	1.122	2.064	2.064	4.232	29.321	4.346	229
Percentage Hispanic (%)	0.260	2.377	3.792	7.092	6.435	91.086	11.018	229
Percentage Black (%)	0.000	0.902	1.679	0.310	3.113	55.220	5.341	229
Percentage White (%)	5.532	82.748	91.016	85.910	94.653	98.959	14.881	229
Percentage English Language Learner (%)	0.000	0.410	0.994	2.550	2.338	30.016	4.411	228
Percentage Reduced-price Lunch Eligible (%)	0.775	2.193	3.743	4.220	6.137	9.905	2.338	229
Percentage Economically Disadvantaged (%)^b^	1.847	11.803	19.300	23.912	31.740	85.957	16.793	229
Log of Median Income	10.43	11.06	11.25	11.26	11.44	12.05	0.30	227
Bachelor’s Degree Rate (%)	11.40	29.32	38.23	40.54	49.42	80.67	15.44	227
Poverty Rate (%)	0.636	3.930	6.824	8.397	11.506	37.943	6.209	227
SNAP Receipt Rate (%)^b^	0.899	3.870	6.018	7.023	8.142	35.674	5.224	227
Single-mom Household Rate (%)	4.52	11.29	13.79	14.36	16.23	43.34	5.28	227
Unemployment Rate (%)	3.424	6.037	6.877	7.109	8.103	14.177	1.745	227

^a^ The test scores are standardized on the national level, thus the standardized scores for Massachusetts do not center at 0

^b^ Economically disadvantaged students in Massachusetts are defined as students who are participating in one or more of the four state-administered programs: the Supplemental Nutrition Assistance Program (SNAP), the Transitional Assistance for Families with Dependent Children (TAFDC), the Department of Children and Families’ (DCF) foster care program, and MassHealth (Medicaid)

Table 4 presents the distribution of the standardized test scores (math and ELA) averaged across grades and the average WLL for the 229 GSDs in MA, from 2010 to 2016. The minimum, 10th percentile, median, 90th percentile, maximum and SD for each variable in each year are shown. The median WLLs have a declining trend in general and the maximum WLLs have a slight increasing trend except for year 2013.

**Table 4:** Distribution of standardized test scores and WLLs across GSDs, 2010–2016, averaged across grades

		2010	2011	2012	2013	2014	2015	2016
Standardized Math Test Score	Min	−0.488	−0.348	− 0.361	−0.346	− 0.390	−0.512	− 0.529
	10th percentile	0.170	0.176	0.168	0.141	0.091	0.049	0.007
	Median	0.505	0.528	0.537	0.545	0.498	0.420	0.411
	Mean	0.508	0.521	0.540	0.541	0.499	0.441	0.430
	90th percentile	0.879	0.888	0.931	0.958	0.922	0.871	0.897
	Max	1.163	1.199	1.360	1.388	1.324	1.246	1.347
	SD	0.278	0.279	0.303	0.318	0.324	0.316	0.345
Standardized ELA Test Score	Min	−0.642	− 0.529	− 0.558	− 0.588	−0.582	− 0.535	−0.449
	10th percentile	0.089	0.125	0.102	0.076	0.093	0.083	0.084
	Median	0.450	0.482	0.481	0.466	0.486	0.479	0.454
	Mean	0.452	0.476	0.476	0.464	0.469	0.467	0.472
	90th percentile	0.854	0.891	0.889	0.903	0.896	0.826	0.896
	Max	1.194	1.115	1.287	1.272	1.227	1.146	1.230
	SD	0.296	0.298	0.307	0.318	0.312	0.303	0.306
90th Percentile WLLs (μg/L)	Min	0	0	0	0	0	0	0
	10th percentile	1.197	1.000	1.278	1.000	1.195	1.000	1.101
	Median	4.000	3.903	3.819	3.122	3.308	3.018	3.300
	Mean	4.760	4.417	4.590	4.203	4.393	4.315	4.452
	90th percentile	8.635	8.000	8.833	8.010	7.859	8.358	7.983
	Max	47.538	46.668	47.830	17.000	50.000	50.000	50.000
	SD	4.376	4.038	4.249	3.044	4.304	4.453	4.681

**Fig. 1:**
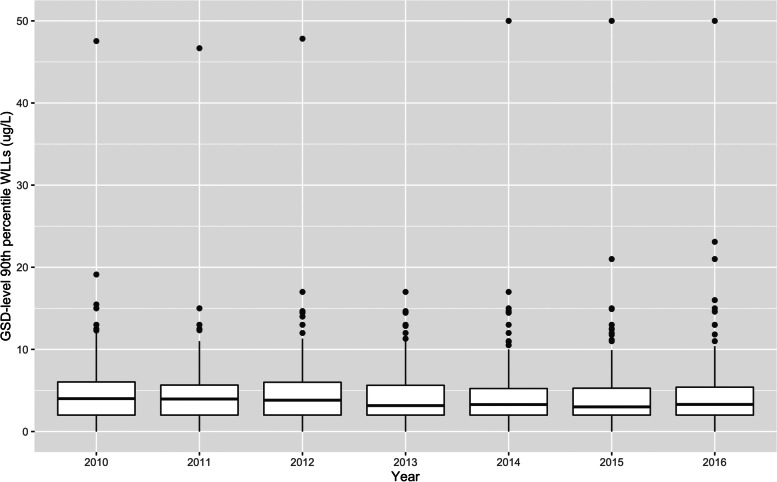
Distribution of GSD-level WLLs (μg/L), 2010–2016

Figure 1 presents the box plot distribution of GSD-level WLLs from 2010 to 2016. Each point represents the WLL level for a GSD-year observation. In each calendar year, some GSDs had WLLs above the 15 μg/L action level. In all years except for year 2013, there was one GSD with WLL over 45 μg/L, much higher than all other GSDs in MA.

**Fig. 2:**
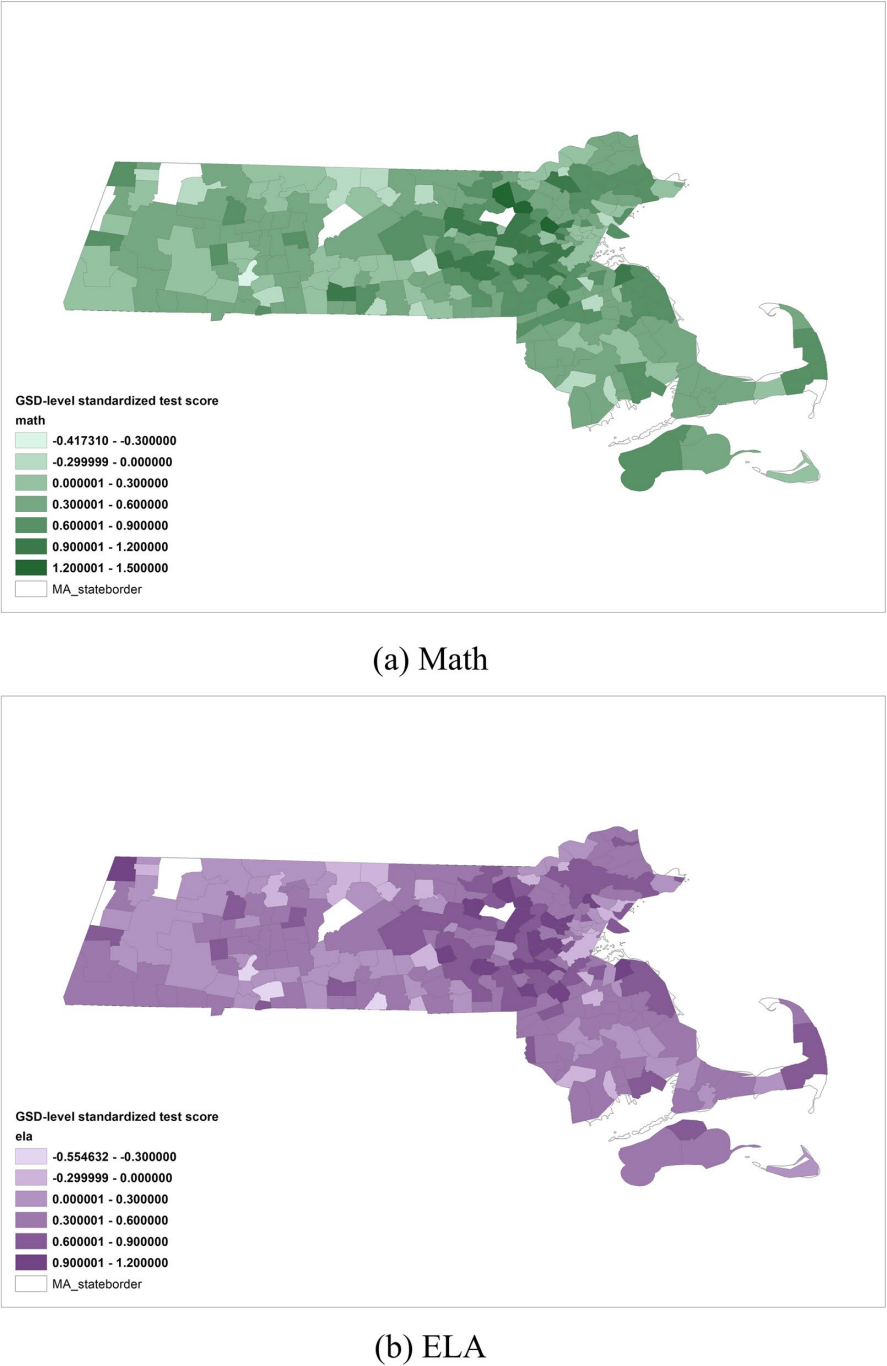
GSD-level math (green) and ELA (purple) test score in Massachusetts, pooled across grades and years

Figure 2 illustrates the GSD-level standardized test scores for both subjects, pooled over 7 years from 2010 to 2016 and 6 grades from grade 3 to grade 8.

Geographic distribution of GSD-level standardized test score was plotted using ArcGIS Pro 2.8 licensed by Harvard University Center for Geographic Analysis. The shapefiles for GSD maps were retrieved from Stanford Education Data Archive (SEDA) version 3.0 [47].

### Regression Results

We investigated two outcomes of interest: the standardized math test scores and the standardized ELA test scores. The main regression results are presented in Table 5. For each outcome of interest, three models are presented. The first one is a crude model with only the exposure of interest, the cohort fixed effects and the time fixed effects. Urbanicity, GSD-level SES characteristics, student racial compositions and student SES characteristics are progressively added to the second and third models. For ease of interpretation, results are presented for a 5 μg/L increase in WLL. After adjusting for all the above-mentioned covariates, the cohort fixed effects and year fixed effects (column 3), a 5 μg/L increase in WLL in a GSD is associated with a 0.00684 SD decrease in the standardized math test score in the GSD in the same year (95% CI: 0.00021, 0.01348). No association between WLL and standardized ELA test score was found. The full regression results with coefficients and the corresponding standard errors for all six models are presented in the Additional file 1. Among the covariates, strong predictors of standardized test score include single-mom household rate, unemployment rate, student racial composition, proportion of ELL and proportion of economically advantaged students. The aim of this study is to obtain an unbiased estimate of the association between WLL and standardized test score, and collinearities among covariates are not precluded. Thus, the coefficients for covariates may be subject to collinearity-related bias.

**Table 5:** Association of GSD-level WLL (per 5 μg/L) in CWSs with standardized test scores

	(1) β (95% CI)		(2) β (95% CI)		(3) β (95% CI)	
	Standardized Math Test Score					
WLL (5μg/L)	− 0.00967***	(− 0.01631, − 0.00302)	− 0.00867***	(− 0.01565, − 0.00228)	−0.00684**	(− 0.01348, − 0.00021)
Observations	8113		8067		8003	
	Standardized ELA Test Score					
WLL (5μg/L)	−0.00450	(− 0.01085, 0.00185)	− 0.00596*	(− 0.01237, 0.00045)	−0.00150	(− 0.00788, 0.00488)
Observations	8112		8067		8003	

All models adjusted for time fixed effects and cohort fixed effects and are weighted on total number of enrollments in each cohort-year, with 5th and 95th percentile weight truncation Model (1) is a crude model. Model (2) adjusted for urbanicity and GSD SES characteristics. Model (3) adjusted for urbanicity, GSD SES characteristics, student racial compositions and student SES characteristics. *p < 0.1, **p < 0.05, ***p < 0.01

### Sensitivity analyses

We performed several sensitivity analyses. First, we compared the final model in Table 5 Column 3 (hereafter referred to as model 3) to alternative models with cohort fixed effects but no time fixed effects, and with time fixed effects but no cohort fixed effects. Both alternative models are nested in model 3, and we used F-tests to compare these models. Model 3 is significantly better than the model with only cohort fixed effects [F _(6, 6752)_ = 56.969; *p* < 0.001], and the model with only time fixed effects [F _(1227, 6752)_ =9.0629; p < 0.001]. We then used the Hausman test to compare the fixed effects model with the random effects model with the same set of covariates; the results suggested that the fixed effects model is preferred over the random effects model [Chi-2 _(17)_ = 586.78; *p* < 0.001] [55].

The standard criteria for evaluating whether adjusting for a variable or a set of variables contributes to confounding control are: (1) the variable is a predictor of the exposure, and (2) the coefficient changes after the variable is adjusted for. To evaluate whether the time fixed effects contributed to confounding control, we first calculated the Spearman’s rank correlation coefficient between WLL and year. We found that WLL in GSDs is negatively correlated with year [r = − 0.049; *p* < 0.001], thus calendar year is a predictor of WLL. We then looked at the regression coefficient for the model with only cohort fixed effects but not time fixed effects: the coefficient for a 5 μg/L change in WLL is − 0.0105, which is different from the coefficient in model 3 (− 0.0068). Thus, time fixed effects should be included in the model for confounding control.

We then evaluated whether cohort fixed effects contributed to confounding control. The result of a Kruskal-Wallis test [Chi-2 _(1250)_ = 6107.7, *p* < 0.001] suggests that cohort is a predictor of WLLs. The regression coefficient for the model with only time fixed effects but not cohort fixed effects is 0.0122 per 5 μg/L increase in WLL, which is significantly different from the coefficient in model 3 (− 0.0069). Thus, cohort fixed effects should also be included in the model for confounding control.

We also looked at models with lagged lead exposure. When the main independent variable in model 3 is replaced with WLL from the previous year, no association is found between lead exposure and standardized math test score [coefficient = − 0.00047, SD =0.00769, *p* = 0.95]. This suggests that WLL is only associated with children’s test scores in the same year and does not have a lag effect on the following year.

### Effect measure modification by grades

**Fig. 3:**
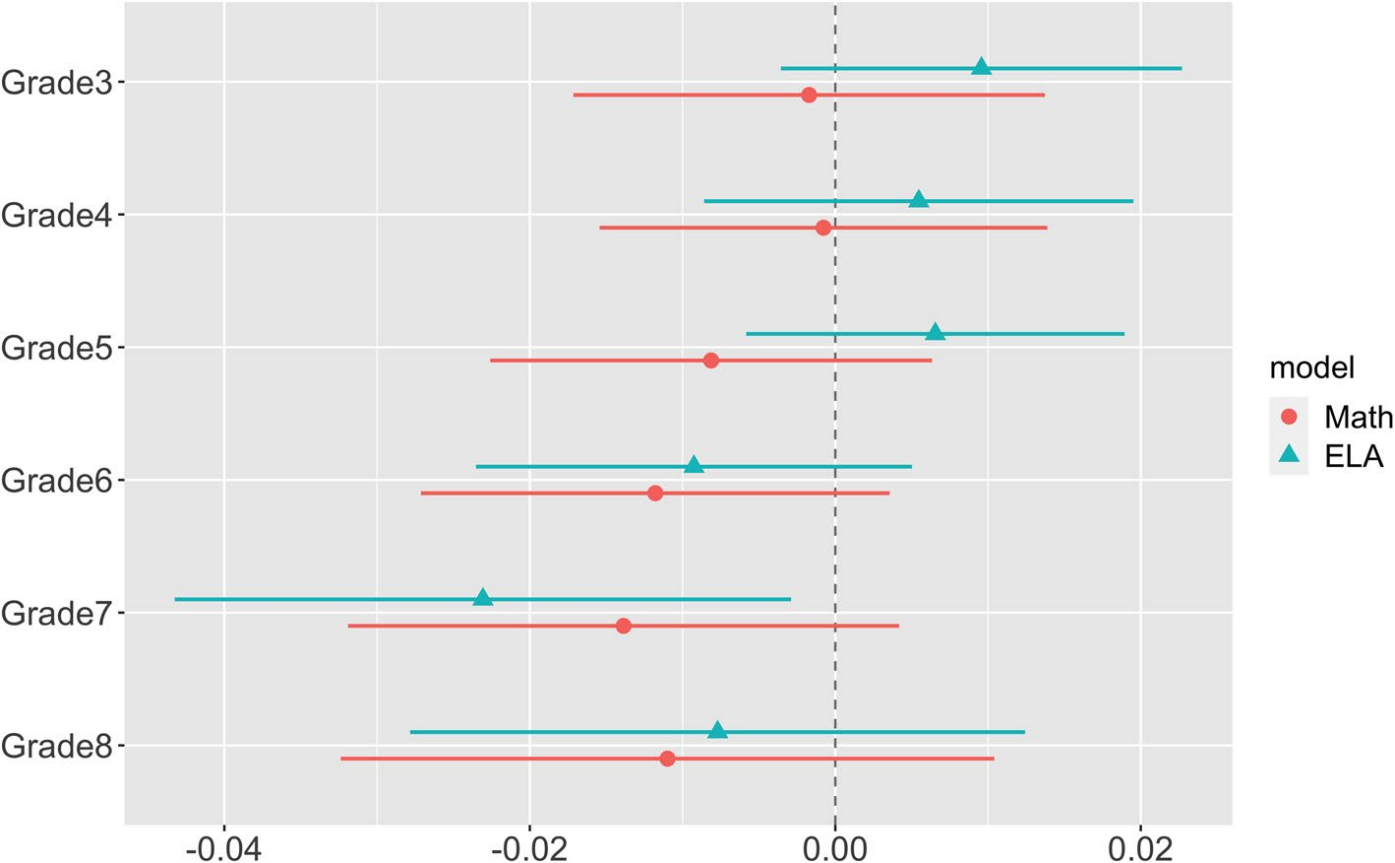
Regression coefficients and 95% confidence intervals for the association between WLLs and standardized test scores

To examine the potential effect modification by grades, we conducted a stratification analysis by grades. The stratified models applied the same covariates, fixed effects and weights as model 3 in Table 5. The regression coefficients and their respective 95% confidence intervals are shown in Fig. 3. The number of observations and the statistical power of each model was reduced after stratification, thus most regression coefficients had 95% confidence intervals covering the null. For both math and ELA, the regression coefficients appear to have a slight decreasing trend as grade increases. We then examined the statistical significance of the effect measure modification by grades using interaction terms between the WLLs and integer grades. The effect modification by grade for math is not statistically significant [coefficient = − 0.00281, standard error = 0.00205, *p* = 0.17]. Because no association between WLL and ELA was found, we did not test for effect modification for ELA.

The models were adjusted for time fixed effects, cohort fixed effects, urbanicity, GSD SES characteristics, student racial composition and student SES characteristics, weighted on total number of enrollments in each cohort-year with 5th and 95th percentile weight truncation. The coefficients were presented for per 5 μg/L increase in WLLs.

### Effect measure modification by racial compositions

We further tested the potential racial disparities of the association. A binary variable was created flagging GSD-grade-year observations with White students lower than 90%. An interaction term between this binary variable and WLL was added to model 3 and the coefficient of this term is tested. The effect measure modification by grade is statistically significant for math [coefficient = − 0.0242, standard error = 0.0051, *p* < 0.01]. We did not test for effect modification for ELA because no association was found between WLL and ELA test score. For cohorts with less than 90% of White students, higher WLLs are associated with a larger reduction in standardized math test scores, compared with cohort years with more than 90% of White students.

## Discussion

Using a multivariate linear regression controlling for school district, grade level, calendar year, urbanicity, racial composition and SES, we found that a 5 μg/L higher WLL was associated with a 0.00684 SD decrease of the average math test score. Because the model has a structure similar to the DID analysis (Table 2), the exact interpretation of this association is that a 5 μg/L increase in GSD-level WLL from last year to the current year is associated with a 0.00684 SD decrease in the average Math test score of the GSD from last year to the current year. We have also found a racial disparity of this association: the association is larger for cohorts with higher proportion of non-White students. We did not find effect modification of this association by grade.

The two-way fixed effects model (fixed effects for both unit and time) has a structure similar to the DID analysis: the DID estimator is proven to be equivalent to the two-way fixed effects model in the two group and two time period setting [51], and equivalent to a weighted two-way fixed effects model in the multi-period setting [53]. However, the causal interpretation of the association found depends on several critical assumptions. The most important assumption is the parallel trend assumption: the time-varying covariates should capture all time-varying factors other than WLL that affect the GSD-level standardized test scores. This assumption is commonly tested by looking at pre-treatment parallel trends [56]. In this study, we could not test this assumption because we cannot observe the trends of student test scores over a few years where WLLs of all GSDs remain constant, thus we cannot credit a causal interpretation of the results. Nevertheless, as analyzed in the sensitivity analysis, adding the cohort and year fixed effects controlled for unmeasured confounders that were constant within cohorts, and common trends across cohorts. For instance, lead in soil and dust is another major source of lead exposure that mainly comes from leaded paint and leaded gasoline, which were banned in the 1970s and 1990s, respectively. Thus, the levels of legacy lead deposition in outdoor soil and indoor dust from old buildings are expected to be relatively constant or follow a common slow decreasing trend across all GSDs in MA over the study period, which are controlled for by design. The two-way fixed effects model, compared with other multivariate linear regression models, provides a closer-to-unbiased estimate of the association between WLL and children’s academic performances.

WLLs are generally low in this cohort, and well within regulatory limits. As shown in Fig. 1 and Table 4, the WLL for most GSDs are below 10 μg/L, and the median WLL level across GSDs has decreased from 4.0 μg/L to 3.3 μg/L from 2010 to 2016. However, the WLL can still be as high as 50 μg/L in some GSDs, as in years 2014, 2015 and 2016. Using estimates from our regression results in Table 5, lead exposure from WLLs of this magnitude is approximately associated with a 0.06835 SD decrease in the math test score, compared with if the lead concentration was at the lowest levels in the state (close to zero). A 50 μg/L higher WLL would move the district’s average math test score from median level in the country to the 47.3rd percentile. The association should only be interpreted on school district levels, not on an individual level.

For the same cohort of students, lead exposure and test score information were matched on years. Based on MA DEP’s lead monitoring protocols, we estimated that about 95.1% of the lead exposure data between 2010 and 2016 were from water samples collected in the Q3 monitoring period (June to October) of each year. The MCAS standardized tests for elementary and secondary schools in MA are mostly administered during April and May [57]. Thus, for a cohort of students in this study, the lead exposure data was likely to be measured a few months after the test score outcomes. This is an important limitation in the analysis-level. We matched the exposure and outcome information in the same years assuming the ranking of the 90th percentile WLL in a GSD does not change much over a few months from April–May to the summer. This assumption is plausible because there is no required measurement of lead concentrations during these months, providing little incentive for the CWSs to change their procedures. The remaining 4.9% of the lead concentration data were measured from CWSs under standard semi-annual monitoring, where the samples were collected during the two monitoring periods Q2 and Q4 spanning across January to December, covering the months before state standard testing. The WLLs in these CWSs can be matched with the standardized test scores in the same year, with no assumption needed.

We found significant association between WLL with math test score, yet no association with ELA test score. This difference is consistent with another study that investigated the relationship between children’s lead exposures and test scores in New York counties: in both bi-variate and multiple regression, the association of BLL with math test score had much higher statistical significance than BLL’s association with ELA score [13]. Other studies have found BLL being associated with both math and ELA test scores with no apparent difference in statistical power [12, 14, 35, 37]. As illustrated in Fig. 3, the associations between WLL and ELA test scores were positive but not significant for children in grades 3 to 5, while negative associations have been detected among children of grades 6 to 8. The combination of slightly positive associations at younger ages and negative associations at older ages resulted in an insignificant association over all ages. The slightly positive association was found between WLL and ELA test score among younger students in this study might be due to chance given the low statistical power (caused by the relatively crude measurement and matching of the exposure), as well as the non-differential misclassification of the exposure that shifts the estimate towards the null, which will be discussed later. It does not exclude the existence of such association.

We tested for lag effects of WLL and children’s test scores and did not find an association between standardized test score and WLL in the previous years. The lack of lagged association indicates that compared with the change in WLL from last year to the current year, the change in WLL from two years before to last year is not as predictive of the change in average test score from last year to the current year. This might be due to the reduction in sample size after lagging that reduced the statistical power: the number of observations for each GSD-grade reduced from 7 to 6 after lagging. Another possibility is that there might be an acute toxicity of lead exposure. This is consistent with a study by Lanphear et al., where they tested the association between early childhood BLL, maximum BLL before IQ measurement, lifetime average BLL and concurrent BLL with child IQ deficit, and found that the concurrent BLL exhibited strongest association with IQ, indicating a potential acute effect of lead toxicity [9]. However, this does not mean that the neurotoxic effect of lead is only short-term. Studies have shown that the majority of lead in human body is stored in the bones with a very slow turnover rate [58] and can re-mobilize into the blood circulation and affect target organs in response to changes of the human body such as pregnancy [59], leading to long-term health impacts.

The implications of a detectable effect of WLLs on academic performance even at the modest levels evident in MA are significant and timely. The detected association between WLLs and math test score, although modest in magnitude, can have life-long impacts on students, such as decreased chances of college admissions and lower lifetime incomes [60, 61]. Cognitive damages associated with lead exposure impose high societal costs [62, 63] and there is good evidence of the efficacy of lead reduction efforts [64], especially for WLLs. Furthermore, EPA has recently undertaken to investigate revisions to the LCR [65]. EPA’s initial proposed revisions may not reduce WLLs and hence, lead exposures [66]; however, improvements in regulatory requirements, enforcement and compliance could produce significant social benefits in the form of reduced lead damages [4, 62, 63, 67].

This study has several limitations. First, the exposure measurement was crude and ecological. WLL data was only available in its 90th percentile form with no additional information on its distribution. We rely on the assumption that PWSs with higher 90th percentile WLLs have higher lead concentrations in general. We also assume that children of grade 3 to 8 would mainly consume water from the CWS supplying their school district. To the extent that they consume water from non-community water supplies or bottled water, our exposure estimates are erroneous. This non-differential misclassification could reduce the detected effect size (closer to the null) compared to the actual effect, as well as compromise statistical power. In addition, the seasonality of the WLL sampling could not be controlled for: First, the sampling collection time was not available, thus the season of sampling of Q2 and Q4 monitoring periods could not be identified. Second, a GSD may be served by multiple CWS with different monitoring periods, thus controlling for monitoring periods for each GSD-year observation was also not feasible. Unable to control for sampling season could potentially produce bias due to the difference in detected WLL between summer and winter, which may be influenced by temperature and disinfection practices. However, as more than 95% of the CWS-level WLLs were collected during Q3 monitoring period (summer), the magnitude of such bias is expected to be minimal. Moreover, restricting to 229 out of 294 GSDs may potentially produce selection bias, yet we found no significant difference between the included and excluded GSDs, minimizing the likelihood of selection bias due to excluded GSDs. Finally, we cannot exclude confounding by omitted time-varying covariates, although we believe there are a limited number of predictors that would be associated with WLL.

## Conclusions

Lead is a neurotoxic agent that contaminates drinking water mainly via the corrosion of plumbing components such as lead pipes and solder in the public and especially residential plumbing systems. Since EPA’s 1991 LCR was issued, concentrations of lead in drinking water may have fallen, and the median WLL levels in MA, which are relatively low, have decreased from 2010 to 2016. Nonetheless, this study suggests that lead from community public water systems may still affect children’s cognitive development, as reflected on their educational achievements. In school districts in MA, higher concentrations of lead in PWSs are associated with lower average math test score in the same year. Reducing math performance may have long-term impacts on children’s academic success and future career options. More efforts are needed to further control lead in drinking water.

## Supplementary Information

**Additional file 1.** Full regression results of GSD-level standardized academic test scores (math and ELA) on WLL and covariates.

**Additional file 2.** (CSV 643 kb)

## Data Availability

The datasets generated and analyzed during the current study are included in this article and its supplementary information files. The 90th percentile lead concentration dataset of Massachusetts community water systems is maintained by the Massachusetts Department of Environmental Protection (DEP). The dataset for the most recent year (2020) is available at https://www.mass.gov/service-details/public-water-systems-90th-percentile-lead-sampling-results. Data from earlier years are also publicly available upon reasonable request to the Massachusetts DEP, with contact information listed in the webpage above. The standardized math and ELA test scores dataset, GSD-level covariates dataset and GSD maps are published by Stanford University at https://exhibits.stanford.edu/data/catalog/db586ns4974 [47].

## Abbreviations

AL: Action Level
BLL: Blood Lead Level
CWS: Community Water System
DEP: Department of Environmental Protection
DID: Difference-in-difference
ELA: English Language Arts
ELL: English Language Learners
EPA: United States Environmental Protection Agency
FERPA: The Family Educational Rights and Privacy Act
GSD: Geographic School District
LCR: Lead and Copper Rule
MASSGIS: Massachusetts Bureau of Geographic Information
MCAS: Massachusetts Comprehensive Assessment System
MWRA: Massachusetts Water Resources Authority
PWS: Public Water System
SD: Standard Deviation
SEDA: Stanford Education Data Archive
SES: Socioeconomic Status
SNAP: Supplemental Nutrition Assistance Program
TAFDC: Transitional Assistance for Families with Dependent Children
WLL: Water Lead Level
WLS: Weighted Least Squares
