# Supplementary material for: Lead contamination of public drinking water and academic achievements among children in Massachusetts: a panel study
Source: BMC Public Health. 2022 Jan 15;22:107. doi: 10.1186/s12889-021-12474-1 (PMC8761345; doi:10.1186/s12889-021-12474-1)
Supplement: Supplementary file 1 — Additional file 1. Full regression results of GSD-level standardized academic test scores (math and ELA) on WLL and covariates. [file 12889_2021_12474_MOESM1_ESM.docx]

| Full regression results of GSD-level standardized academic test scores (math and ELA) on WLL and covariates | | | | | | |
| --- | --- | --- | --- | --- | --- | --- |
|  | Math | | | ELA | | |
|  | (1) β (se) | (2) β (se) | (3) β (se) | (1) β (se) | (2) β (se) | (3) β (se) |
| WLL (5ug/L) | -0.00967*** | -0.01793*** | -0.00684** | -0.00450 | -0.00696* | -0.00150 |
|  | (0.00339) | (0.00682) | (0.00339) | (0.00324) | (0.00327) | (0.00326) |
| Urban |  | 0.023 | 0.022 |  | 0.019 | 0.024 |
|  |  | (0.021) | (0.021) |  | (0.020) | (0.020) |
| Town |  | 0.019 | 0.018 |  | -0.032 | -0.029 |
|  |  | (0.030) | (0.030) |  | (0.029) | (0.029) |
| Rural |  | 0.015 | 0.016 |  | 0.004 | 0.005 |
|  |  | (0.010) | (0.010) |  | (0.010) | (0.010) |
| Log of median income |  | 0.048 | -0.026 |  | 0.002 | -0.062 |
|  |  | (0.044) | (0.044) |  | (0.042) | (0.042) |
| Bachelor's degree rate |  | 0.404*** | 0.447*** |  | 0.013 | 0.103 |
|  |  | (0.109) | (0.108) |  | (0.105) | (0.104) |
| Poverty rate |  | -0.096 | -0.105 |  | 0.094 | 0.059 |
|  |  | (0.069) | (0.068) |  | (0.066) | (0.066) |
| SNAP receipt rate |  | -0.387*** | -0.375** |  | 0.298** | -0.032 |
|  |  | (0.141) | (0.146) |  | (0.135) | (0.141) |
| Single-mom household rate |  | -0.249** | -0.270** |  | -0.540*** | -0.510*** |
|  |  | (0.117) | (0.116) |  | (0.112) | (0.111) |
| Unemployment rate |  | 1.015*** | 1.261*** |  | 0.338* | 0.719*** |
|  |  | (0.202) | (0.202) |  | (0.194) | (0.194) |
| Proportion of Native American student |  |  | 0.702 |  |  | -0.099 |
|  |  |  | (0.686) |  |  | (0.662) |
| Proportion of Asian student |  |  | 0.994*** |  |  | 0.240** |
|  |  |  | (0.127) |  |  | (0.122) |
| Proportion of Hispanic student |  |  | -0.240** |  |  | 0.001 |
|  |  |  | (0.109) |  |  | (0.105) |
| Proportion of Black student |  |  | -0.600*** |  |  | -0.186 |
|  |  |  | (0.171) |  |  | (0.164) |
| Proportion of English Language Leaner |  |  | -0.420** |  |  | -0.366** |
|  |  |  | (0.185) |  |  | (0.178) |
| Proportion of reduced-price lunch eligible student |  |  | 0.139 |  |  | -0.220 |
|  |  |  | (0.157) |  |  | (0.151) |
| Proportion of economically disadvantaged student |  |  | -0.449*** |  |  | -0.508*** |
|  |  |  | (0.040) |  |  | (0.039) |
| Observations | 8,113 | 8,067 | 8,003 | 8,112 | 8,067 | 8,003 |
| R2 | 0.001 | 0.011 | 0.034 | 0.0001 | 0.001 | 0.020 |
| Adjusted R2 | -0.180 | -0.169 | -0.145 | -0.181 | -0.181 | -0.161 |
| Time fixed effects and cohort fixed effects are adjusted in all six models. Standard errors of coefficients are presented in parentheses. | | | | | | |
| *p<0.1; **p<0.05; ***p<0.01 | | | | | | |
